# Use of Digital Technology Tools to Characterize Adherence to Prescription-Grade Omega-3 Polyunsaturated Fatty Acid Therapy in Postmyocardial or Hypertriglyceridemic Patients in the DIAPAsOn Study: Prospective Observational Study

**DOI:** 10.2196/37490

**Published:** 2022-07-25

**Authors:** Gregory P Arutyunov, Alexander G Arutyunov, Fail T Ageev, Tatiana V Fofanova

**Affiliations:** 1 Department of Internal Medicine Pirogov Russian National Research Medical University Moscow Russian Federation; 2 Department of Outpatient Medical and Diagnostic Technologies of the Research Institute of Cardiology named after A.L. Myasnikov Federal State Budgetary Institution National Medical Research Center of Cardiology named after Academician E.I. Chazov Ministry of Health of the Russian Federation Moscow Russian Federation

**Keywords:** primary care, research, myocardial infarction, cardiology, heart, cardiac, cardiac health, digital health, electronic patient engagement, eHealth, patient engagement, clinical report, treatment, treatment adherence

## Abstract

**Background:**

Maintaining sustained adherence to medication for optimal management of chronic noninfectious diseases, such as atherosclerotic vascular disease, is a well-documented therapeutic challenge.

**Objective:**

The DIAPAsOn study was a 6-month, multicenter prospective observational study in the Russian Federation that examined adherence to a preparation of highly purified omega-3 polyunsaturated fatty acids (Omacor) in 2167 adult patients with a history of recent myocardial infarction or endogenous hypertriglyceridemia.

**Methods:**

A feature of DIAPAsOn was the use of a bespoke electronic patient engagement and data collection system to monitor adherence. Adherence was also monitored by enquiry at clinic visits. A full description of the study’s aims and methods has appeared in JMIR Research Protocols.

**Results:**

The net average reduction from baseline in both total and low-density lipoprotein cholesterol was approximately 1 mmol/L and the net average increase in high-density lipoprotein cholesterol was 0.2 (SD 0.53) mmol/L (*P*<.001 for all outcomes vs baseline). The mean triglyceride level was 3.0 (SD 1.3) mmol/L at visit 1, 2.0 (SD 0.9) mmol/L at visit 2, and 1.7 (SD 0.7) mmol/L at visit 3 (*P*<.001 for later visits vs visit 1). The percentage of patients with a triglyceride level <1.7 mmol/L rose from 13.1% (282/2151) at baseline to 54% (1028/1905) at the end of the study. Digital reporting of adherence was registered by 8.3% (180/2167) of patients; average scores indicted poor adherence. However, a clinic-based enquiry suggested high levels of adherence. Data on health-related quality of life accrued from digitally engaged patients identified improvements among patients reporting high adherence to study treatment, but patient numbers were small.

**Conclusions:**

The lipid and lipoprotein findings indicate that Omacor had nominally favorable effects on the blood lipid profile. Less than 10% of patients enrolled in DIAPAsOn used the bespoke digital platform piloted in the study, and the level of self-reported adherence to medication by these patients was also low. Reasons for this low uptake and adherence are unclear. Better adherence was recorded in clinical reports.

**Trial Registration:**

ClinicalTrials.gov NCT03415152; https://clinicaltrials.gov/ct2/show/NCT03415152

## Introduction

Non–high-density lipoprotein cholesterol (non-HDL-C) blood lipids are a source of residual cardiovascular risk in patients whose low-density lipoprotein cholesterol (LDL-C) levels are well controlled by medication, primarily statins [1-9].

Optimal risk reduction in cardiovascular disease, as in other forms of major noncommunicable disease, depends substantially on patients continuing to take their medications for extended periods of time. This can be a particular challenge in conditions such as hyperlipidemia, where the connection between symptomless elevations of blood lipid levels and major cardiovascular events can seem abstract or remote [10,11].

Recent comparative research in Russia and Norway has disclosed poor attainment of cholesterol targets in both countries, despite a notably higher prescription rate of these drugs in Norway [12]. Suboptimal patient adherence to prescribed treatments is likely to be a contributor to such findings, which illustrates that the challenges of promoting and sustaining adherence to therapy are not confined to any one country. It is nevertheless clear from the results of the CEPHEUS (Centralized Pan-Russian Survey of the Undertreatment of Hypercholesterolemia) II study that failure to reach targets for lipid-based risk reduction is widespread in Russia [13]. Patient-related factors associated with nonattainment of targets identified in that study included the consideration that it was acceptable to miss prescribed doses more than once per week. Poor adherence to medication for hypertension has likewise been documented in the Izhevsk Family Study II [14].

Those findings exemplify observations that the rates of both discontinuation and nonadherence to therapy are uniformly high in clinical trials of lipid-lowering drugs and even higher in unselected populations, with adherence deteriorating in proportion to the duration of follow up [15]. Analysis of a large Swiss health care claims database (N=4349) revealed that overall adherence to drug therapy for secondary cardiovascular prevention after myocardial infarction (MI) was only moderate, but that patients with high adherence to lipid-lowering therapy had a significantly reduced risk for all-cause mortality and major cardiovascular events, illustrating the potential for improvement of longer-term outcomes [16].

Omega-3-acid ethyl esters (OM3EE) are available as a prescription-only medication (Omacor, Abbott Laboratories GmbH) that is a preparation of highly purified long-chain omega-3 polyunsaturated fatty acids (n-3 PUFAs) (eicosapentaenoic acid/docosahexaenoic acid in a 1.2:1 ratio and 90% purity); this medication is widely approved for use at a daily dose of 1 g for the secondary prevention of major cardiovascular events in patients who have survived an MI, or at doses of 2 to 4 g/day for the regulation of triglyceride (TG) level. Prescription-only n-3 PUFAs such as OM3EE are qualitatively distinct from dietary n-3 PUFA supplements and have been evaluated in a range of clinical trials [17,18].

The emergence of widely available digital and internet technologies with the potential to provide immediate bidirectional communication between health care professionals (ie, doctors, nurses, and pharmacists) and patients may be an important new resource for promoting long-term adherence to therapies [19]. The DIAPAsOn study was devised to explore patient adherence to OM3EE therapy through the medium of digital technology tools [20].

## Methods

### Overview

A comprehensive description of the methodology of the DIAPAsOn study has previously been published, including baseline demographic data [20]. Briefly, DIAPAsOn was a prospective observational study conducted at >100 centers in the Russian Federation that was devised to examine adherence to a prescription of OM3EE as either a secondary preventive medical therapy (at a dose of 1 g/day) for patients with a history of recent MI or for blood lipid regulation (at a dose of 2-4 g/day) in patients with endogenous hypertriglyceridemia insufficiently responsive to dietary modification or drug therapy.

Participants were required to be adults (aged ≥18 years) with a history of MI for whom OM3EE was prescribed as part of a secondary prevention strategy; to have Fredrickson endogenous type IIb or III hypertriglyceridemia not satisfactorily controlled by statin therapy; or to have Fredrickson endogenous type IV hypertriglyceridemia not sufficiently controlled by a lipid-moderating diet. In addition, the included patients took OM3EE for less than 2 weeks prior to enrolment. DIAPAsOn is registered at ClinicalTrials.gov (NCT03415152).

### Schedule of Visits and Data Collection

The DIAPAsOn study had a scheduled duration of 6 months. Clinic visits were scheduled at the start of the study (visit 1), at approximately 3 months (visit 2), and at the end of the study (visit 3). At each of the 3 scheduled clinic visits, patients were questioned about their compliance with the OM3EE therapy using the Questionnaire of Treatment Compliance [21]. This instrument, which has been used in Russia to investigate compliance with other cardiovascular medications, produces a numerical indication of compliance, as follows: 12 to 15 points, very high; 8 to 11 points, high; 4 to 7, moderate; and 0 to 3, low.

A blood lipid profile was determined at each visit, and blood pressure and heart rate data were collected. Adverse events and hospitalization were recorded. Patients also received intervisit phone calls focused on adherence to therapy and safety.

A central aspect of DIAPAsOn was the use of remote digital technology that allowed patients to submit data and report on matters such as health-related quality of life (HRQoL) and product usability (rated as very good, good, moderate, or poor).

The electronic patient engagement and data collection system used in DIAPAsOn was developed in collaboration with the medical online platform Rosmed.info, which has wide-ranging experience in the development and operation of mobile health applications in the Russian Federation. A fuller description of the system used in DIAPAsOn is featured in a separate paper on the study’s methodology [20].

### Ethics Approval

Ethical oversight of the DIAPAsOn study was exercised by the independent Interuniversity Ethics Committee Gagarinsky pereulok, 37, Moscow, Russian Federation (Protocol No. 09-17 of the Interuniversity Ethics Committee, dated 10/19/2017 and later amendments). All aspects of the DIAPAsOn study, including the associated mobile health app, conformed to relevant national and international legal and ethical regulations and requirements for the conduct of clinical research in human subjects, followed the provisions of the Declaration of Helsinki, and included patients’ right to decline further participation in DIAPAsOn at any time and for any reason, whether stated or not, without prejudice to their subsequent treatment. A list of center investigators appears in Multimedia Appendix 1.

### Statistical Methods

Methods were predominantly descriptive, conducted in accordance with the preapproved statistical analysis plan, and used the statistical programming language R (version 3.4.3).

The primary endpoint—adherence to therapy with OM3EE in post-MI patients or patients with hypertriglyceridemia—was assessed in an analysis population, defined as those patients for whom data were obtained at least at visits 1 and 2.

Analysis of the primary endpoint included determination at the end of the study (ie, visit 3) of the mean adherence rate, which was defined as the number of days for which the patient took the full prescribed dose of OM3EE during the specified period divided by the total number of days in that period. The mean score on the National Questionnaire of Treatment Compliance was calculated at the same time.

Comparison of individual patient data between visits was based on either a 2-tailed Student *t* test (for dependent variables) or the McNemar test (for qualitative data).

## Results

### Population Accounting

**Figure 1:**
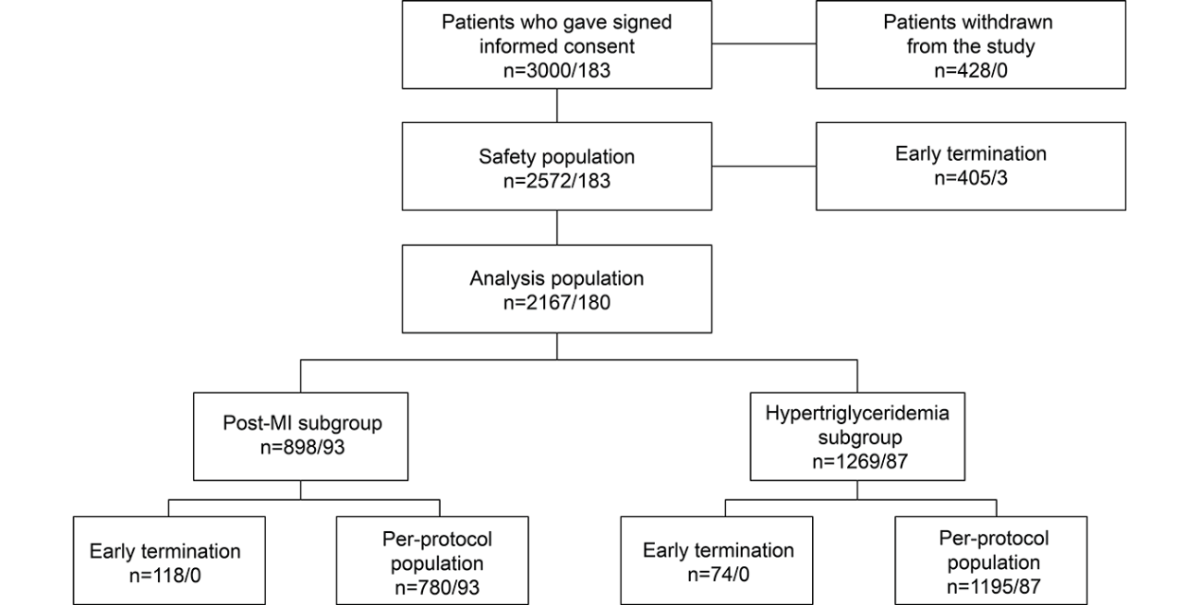
Patient subsets in the DIAPAsOn study. Numbers in the form “xx/yy” indicate patients who did not/did use the study’s digital tools.

A total of 3000 patients were initially included in the program, but 428 (14.3%) were excluded because visit 1 data were incomplete. Valid and complete data from visit 1 were available for 2572 patients (85.7%), who constituted the safety population. After the exclusion of 405 patients lost before visit 3, an analysis population of 2167 patients remained, representing 72.2% of the total enrolled patients (Figure 1). Of these 2167 patients, 898 (41.4%) were taking OM3EE for secondary prevention after an MI and 1269 (58.6%) were taking OM3EE for hypertriglyceridemia.

DIAPAsOn was completed per protocol by 1975 patients (post-MI subgroup, 780/1975; hypertriglyceridemia subgroup, 1195/1975). This was an almost wholly White population (2118/2167, 97.7%) with a near-equal sex distribution (1145 men of 2167 patients, 52.8%; 1022 women of 2167 patients, 47.2%), an average age of 60 years, and an average body mass index of 30 kg/m^2^. There were more men than women in the post-MI subgroup (608/898, 67.7%), whereas women outnumbered men in the hypertriglyceridemia subgroup (732/1269, 57.7%). Investigator-assessed clinically significant abnormalities in systolic and diastolic blood pressure were recorded in 21.4% (463/2167) and 12.8% (277/2167) of patients, respectively.

As illustrated in Figure 1, 180 of the 2167 patients in the analysis population (8.3%) submitted data via the mobile health platform, of whom 93 were enrolled in DIAPAsOn on the basis of a previous MI and 87 on the basis of a diagnosis of qualifying hyperlipidemia. From start to finish, 3 of the initial grouping of 183 patients who used the mobile health platform (1.6%) were withdrawn from the study or discontinued it, compared with 1025 of 2817 (36.4%) of those who did not submit data via the mobile platform. After establishment of the analysis population, early termination rates were 0% and 9.7% for those who did and did not use the mobile platform, respectively (Figure 1).

### Compliance With OM3EE

**Figure 2:**
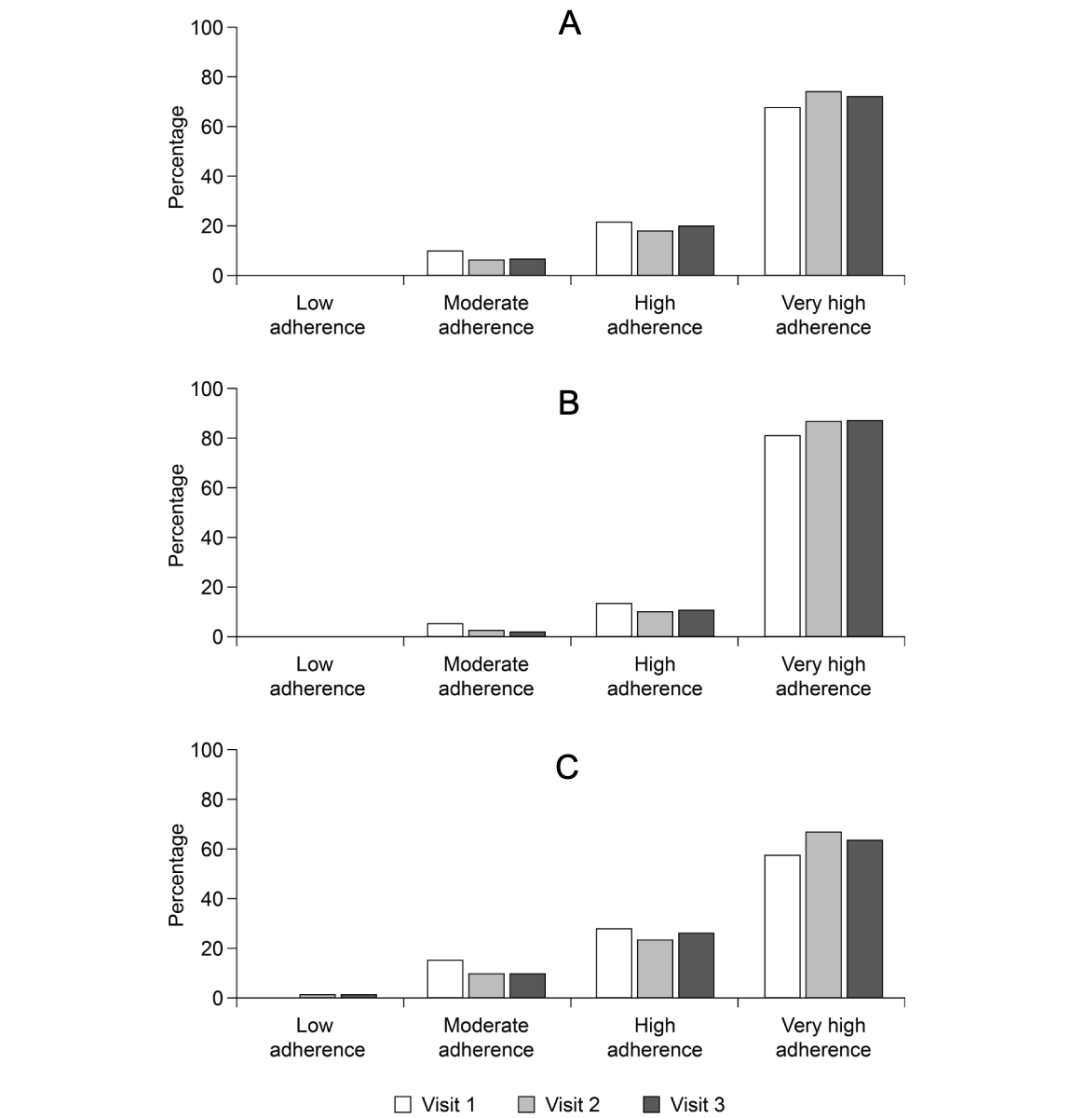
Distribution of adherence categories for (A) the overall population (B), the post–myocardial infarction subgroup, and (C), the hypertriglyceridemia subgroup, based on responses to the National Questionnaire of Treatment Compliance.

The mean duration of OM3EE administration was 166.5 (SD 70.6) days (median 199 days; range 14-268 days). Among the 2167 patients whose compliance was monitored at clinical visits but not self-reported via the mobile app, the mean score on the National Questionnaire of Treatment Compliance at visit 3 was 13 (SD 3) points, signifying high overall compliance with therapy. Mean scores >12 points were also recorded at visit 1 (12.5 points, SD 3.1) and visit 2 (13 points, SD 2.9). Relative to the mean score at visit 1, the mean scores at both visits 2 and 3 were statistically significantly higher (*P*<.001) (Table 1). The distribution of adherence categories for the total study population and for the two subpopulations of DIAPAsOn is shown in Figure 2. Overall, high or very high compliance was recorded for 76/780 (87.9%) of respondents in the post-MI group and 1078/1195 (90.2%) of respondents in the hypertriglyceridemia group. Within the post-MI group, adherence fell significantly at age >75 years (61/81, 75%) compared to all younger age deciles (618/699, 88.4%; *P*=.007, chi-square test).

Very high adherence was reported significantly more often by men than women, especially in the hypertriglyceridemia subset (346/511, 67.7% vs 413/684, 60.4%, respectively; *P*=.007, chi-square test). Among patients with hypertriglyceridemia, very high adherence was also significantly more likely in those who were recorded as not working than those who were working (333/481, 69.2% vs 426/714, 59.7%, respectively; *P*<.001, chi-square test). Adherence was much higher among early school leavers than in any other category of education but, especially in the hypertriglyceridemia subset, this finding was based on small numbers (n=3).

A total of 69/2572 patients (2.68%) discontinued OM3EE during the study. Of these 69 patients, the largest groups cited inconvenience of use (n=11) and reported absence of stock at pharmacies (n=6). A total of 50 patients discontinued use for a variety of other reasons, including the cost of the medication, reluctance to commit to long-term medication discontinued by another physician, normalization of blood lipid values, and change of residence. Inconvenience of use was more often recorded in hypertriglyceridemia patients than post-MI patients (10/52, 19% vs 1/17, 6%, respectively).

Among the 180 patients who registered data via the DIAPAsOn mobile platform, adherence to therapy, expressed as the ratio of days when the full prescribed dose of OM3EE was taken to the total number of days in the treatment period, averaged 0.37 (SD 0.38) over the entire program, corresponding to a low level of adherence. Mean adherence between visits 1 and 2 was 0.48 (SD 0.4), while mean adherence between visits 2 and 3 was 0.24 (SD 0.4; *P*<.001). Between visits 1 and 2, 50% (90/180) of patients had low adherence (<0.5), 15.6% (28/180) had moderate adherence (0.5-0.7) and 34.4% (62/180) had high adherence (≥0.8). Between visits 2 and 3, the proportion of patients with low adherence increased to 75% (135/180), while the proportion with high adherence decreased to 20.6% (37/180). When the data were stratified by adherence level, there was an essentially binary split, with most patients reporting either low adherence (132/180, 74.4%) or high adherence (45/180, 25%).

In the subgroup of 93 patients taking OM3EE for secondary prevention after MI and self-reporting adherence via the study app, mean adherence between visits 1 and 3 was 0.47 (SD 0.39), with 67% (62/93) of patients recording low adherence and 32% (30/93) high adherence. Mean adherence in the post-MI subgroup reached 0.6 (SD 0.38) at visit 2, while at visit 3 it had decreased to 0.33 (SD 0.45; *P*<.001). At visit 2, 32% (30/93) of patients had low adherence (<0.5) and 45% (42/93) had high adherence (≥0.8). By visit 3, the proportion of patients with low adherence had increased to 68% (63/93), while the proportion with high adherence had declined to 30% (28/93).

Among the 87 app-using patients taking OM3EE for hypertriglyceridemia, mean adherence between visits 1 and 3 was 0.25 (SD 0.33), with most patients (83%, 72/87) self-reporting low adherence, and 17% (15/87) recording high adherence. In this subgroup, 69% of patients (60/87) had low adherence (<0.5) at visit 2 and 23% (20/87) had high adherence (≥0.8). By visit 3, these percentages had changed to 83% (72/87) and 10% (9/87), respectively.

Cross-referencing of the results for the National Questionnaire of Treatment Compliance administered at the clinic visits with self-reported adherence, based on the ratio of administered and prescribed dose, established that among patients identified by their response to the National Questionnaire as having very high, high, or moderate adherence to therapy, app-reported mean adherence for the time period between visits 1 and 2 was 50.04%, 52.85% and 24.58%, respectively, while between visits 2 and 3 adherence was 28.67% for those assessed as having very high adherence, 19.11% for those with high adherence, and 5.57% for those with moderate adherence.

In the app-using analysis population as a whole, 64.5% of patients (69/107) rated the usability of OM3EE after 1 month of treatment as very good. A further 29.9% (32/107) and 5.6% (6/107), respectively, rated usability as good or moderate. No patient rated usability as poor. All the patients prescribed OM3EE for secondary prevention post-MI rated the usability as very good (41/58, 71%) or good (17/58, 29%), while among patients treated for hypertriglyceridemia, the usability of OM3EE was rated as very good by 57% of patients (28/49), good by 31% (15/49), and moderate by 12% (6/49).

**Table 1:** Changes in mean score on the National Questionnaire of Treatment Compliance between visit 1 (baseline) and visit 2 (at 3 months) or visit 3 (at study completion, after 6 months).

	Mean score (SD)	*P* value
Visit 1	12.5 (3.11)	N/A^a^
Visit 2	12.99 (2.88)	N/A
Visit 3	12.9 (2.99)	N/A
Visit 2 vs visit 1	0.47 (2.46)	<.001
Visit 3 vs visit 1	0.44 (2.52)	<.001

^a^N/A: not applicable.

### Lipid Indices

At baseline (N=2167), investigator-classified clinically significant deviations from normal for total cholesterol (TC), LDL-C, high-density lipoprotein cholesterol (HDL-C), and TG were recorded in 46.1% (999/2167), 40.9% (887/2167), 14.4% (312/2167) and 65% (1408/2167) of patients, respectively. Mean values for TC, TG, LDL-C, HDL-C and non-HDL-C were 5.55 (SD 1.39) mmol/L, 2.99 (SD 1.29) mmol/L, 3.5 (SD 1.25) mmol/L, 1.27 (SD 0.46) mmol/L, and 4.29 (SD 1.47) mmol/L, respectively.

**Figure 3:**
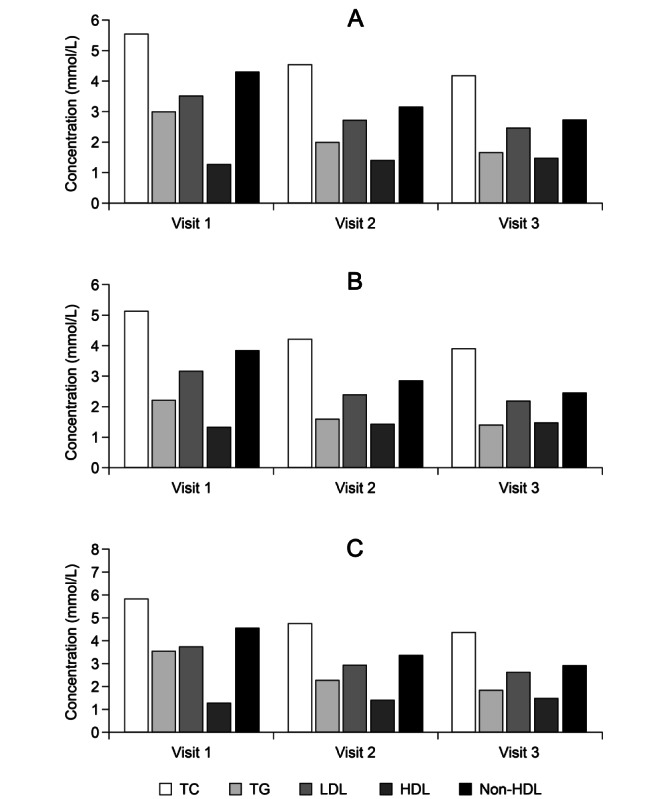
In-study changes in blood lipids in (A) the overall population (B), the post–myocardial infarction subgroup, and (C), the hypertriglyceridemia subgroup. TC: total cholesterol; TG: triglyceride; LDL: low-density lipoprotein; HDL: high-density lipoprotein.

In-study changes in mean blood lipid levels are shown in Figure 3 for the overall DIAPAsOn cohort and for the two subpopulations differentiated by indication. Analysis of lipid profiles stratified by baseline TG status revealed that an increasing TG level was associated with changes in TC that were potentially deleterious to cardiovascular health (Table 2).

**Table 2:** Baseline lipid profile of the analysis population, stratified by triglyceride status, identified progressively more atherogenic patterns of total and lipoprotein cholesterol as triglyceride level increased.

TG^a^ level		TC^b^ (mmol/L)	TG (mmol/L)	LDL-C^c^ (mmol/L)	HDL-C^d^ (mmol/L)	Non-HDL-C (mmol/L)
**Low (<1.7 mmol/L)**						
	Patients, n	282	282	282	282	280
	Mean (SD)	4.67 (1.33)	1.21 (0.31)	2.93 (1.14)	1.33 (0.5)	3.37 (1.4)
	Median	4.5	1.2	2.7	1.2	3.25
**Moderate (1.7-2.3 mmol/L)**						
	Patients, n	431	431	430	431	431
	Mean (SD)	5.31 (1.24)	2.04 (0.19)	3.39 (1.3)	1.31 (0.51)	4 (1.4)
	Median	5.5	2.01	3.1	1.2	4.01
**High (>2.3 mmol/L)**						
	Patients, n	1438	1438	1438	1438	1437
	Mean (SD)	5.79 (1.37)	3.63 (1.08)	3.65 (1.22)	1.25 (0.44)	4.55 (1.42)
	Median	5.9	3.4	3.5	1.1	4.6

^a^TG: triglyceride.

^b^TC: total cholesterol.

^c^LDL-C: low-density lipoprotein cholesterol.

^d^HDL-C: high-density lipoprotein cholesterol.

Lipid profile parameters were recorded at baseline (visit 1), as well as after 3 months (visit 2) and 6 months (visit 3) of follow up. Mean TC at visit 1 was 5.55 (SD 1.39) mmol/L. This had decreased to 4.54 (SD 1.04) mmol/L (*P*<.001) by visit 2, and at visit 3 had been further reduced to 4.17 (SD 1.04) mmol/L (*P*<.001). Across the period of observation, the net average change in mean TC was thus –1.32 (SD 1.28) mmol/L.

At visit 2, mean LDL-C was 2.71 (SD 0.94) mmol/L, an average reduction from visit 1 of 0.77 (SD 0.92) mmol/L (*P*<.001). Further reduction was observed at visit 3, when the mean LDL-C level was 2.46 (SD 0.76) mmol/L (*P*<.001 vs visit 1). The average net decrease in LDL-C was thus 1.02 (SD 1.02) mmol/L.

Mean HDL-C levels at visits 1, 2, and 3 were, respectively, 1.27 (SD 0.46) mmol/L, 1.41 (SD 0.42) mmol/L (*P*<.001 vs visit 1), and 1.44 (SD 0.42) mmol/L (*P*<.001 vs visit 1), with an average increase of 0.2 (SD 0.53) mmol/L over the period of observation.

Non-HDL-C declined by an average of 1.6 (SD 1.54) mmol/L during the period of observation, falling from 4.27 (SD 1.47) mmol/L at visit 1 to 3.14 (SD 1.12) mmol/L at visit 2 (*P*<.001 vs visit 1) and to 2.71 (SD 1.0) mmol/L at visit 3 (*P*<.001 vs visit 1).

Mean TG level was 3.0 (SD 1.3) mmol/L at visit 1, 2.0 (SD 0.9) mmol/L at visit 2, and 1.7 (SD 0.7) mmol/L at visit 3 (*P*<.001 for both vs visit 1). The overall average reduction in the mean TG level was thus 1.32 (SD 1.15) mmol/L across the observation period.

Trends in overall TG levels during DIAPAsOn are displayed in more detail in Table 3, with patients assigned to 1 of 3 baseline distribution categories. Statistically significant changes in the distribution toward lower levels of TG were apparent at both visits 2 and 3, with the percentage of patients recorded as having TG <1.7 mmol/L increasing from 13.1% (282/2151) at baseline to 54% (1028/1905) at the conclusion of the study period, while the percentage recorded as having TG >2.3 mmol/L fell from 66.9% (1438/2151) to 10.4% (198/1905).

**Table 3:** Trends in overall triglyceride levels during the DIAPAsOn study, stratified by baseline triglyceride category.

Baseline triglyceride level, n (%)	Visit 1 (N=2151)	Visit 2 (N=2037)	Visit 3 (N=1905)	*P* value (visit 2 vs 1)	*P* value (visit 3 vs 1)
Low (<1.7 mmol/l)	282 (13.1)	787 (38.6)	1028 (54)	<.001	<.001
Moderate (1.7-2.3 mmol/l)	431 (20)	670 (32.9)	679 (35.6)	<.001	<.001
High (>2.3 mmol/l)	1438 (66.9)	580 (28.5)	198 (10.4)	<.001	<.001

The average differences in TC, LDL-C, and non-HDL-C between baseline and visit 3 were a function of baseline TG. Thus, the reductions in patients with initial TG >2.3 mmol/L were –1.47, –1.1, and –1.7 mmol/L, respectively, while in patients with initial TG 1.7 to 2.3 mmol/L, the average intrastudy reductions from baseline to visit 3 were –1.16, –0.99, and –1.32 mmol/L, respectively. In patients with initial TG <1.7 mmol/L, the average reductions in TC, LDL-C, and non-HDL-C were –0.83, –0.63 and –0.94 mmol/L, respectively. Nevertheless, the change in each parameter in each of these subgroups was statistically significant (*P*<.001). The mean increase in HDL during observation versus baseline was 0.24 mmol/L in patients with TG >2.3 mmol/L (*P*<.001), 0.16 mmol/L in patients with TG 1.7 to 2.3 mmol/L (*P*<.001), and 0.09 mmol/L in patients with TG <1.7 mmol/L (*P*=.002). A statistically significant decrease in TG at visit 3 versus baseline was observed only in the subgroups of patients with baseline TG >2.3 or 1.7 to 2.3 mmol/L (–1.81 and –0.55 mmol/L, respectively; *P*<.001 for both subgroups).

Subanalysis of the patients being treated for hypertriglyceridemia stratified according to the concomitant use or nonuse of statins or fibrates identified no substantial or significant intergroup differences in baseline levels of blood lipid components.

Subsequent in-study trends in blood lipid fractions in both these subgroups are summarized in Table 4 and indicate significant longitudinal trends in both subgroups (*P*<.001 for all indices in both comparisons) with slightly more pronounced responses in patients who were taking additional lipid-regulating drugs in combination with OM3EE. Formal tests for differences between subgroups depending on the use or nonuse of statins or fibrates were not conducted.

Changes between baseline and visit 3 in the 180 patients who registered self-reported adherence in the study mobile app identified no correlations or associations between the level of adherence and absolute changes in levels of lipids or lipoproteins.

The results of investigations into the relationship between rates of adherence and patient demographic factors for the whole analysis population are summarized in Multimedia Appendix 2.

**Table 4:** Trends in lipid and lipoprotein fractions in patients enrolled in the analysis population of DIAPAsOn for hypertriglyceridemia and receiving or not receiving concomitant statins or fibrates.

Change			TC^a^ (mmol/l)		TG^b^ (mmol/l)		LDL-C^c^ (mmol/l)		HDL-C^d^ (mmol/l)	Non–HDL-C (mmol/l)
**Patients receiving statins or fibrates**										
	Visit 3 vs visit 1, mean (SD)	–1.5 (1.3)		–1.7 (1.2)		–1.2 (1.1)		0.2 (0.5)		–1.8 (1.5)
	Visit 3 vs visit 1, median	–1.44		–1.7		–1.0		0.2		–1.9
	*P* value (visit 3 vs visit 1)	<.001		<.001		<.001		<.001		<.001
**Patients not receiving statins or fibrates**										
	Visit 3 vs visit 1, mean (SD)	–1.4 (1.3)		–1.5 (1.1)		–0.84 (0.9)		0.3 (0.6)		–1.7 (1.6)
	Visit 3 vs visit 1, median	–1		–1.32		–0.7		0.2		–1.3
	*P* value (visit 3 vs visit 1)	<.001		<.001		<.001		<.001		<.001

^a^TC: total cholesterol.

^b^TG: triglyceride.

^c^LDL-C: low-density lipoprotein cholesterol.

^d^HDL-C: high-density lipoprotein cholesterol.

### HRQoL Outcomes

HRQoL data accrued from patients who contributed to the digital data collection element of DIAPAsOn are summarized in Table 5. These data represent mean (SD) scores from the the 36-item Short-Form Health Survey (SF-36), which has 8 domains: general health, physical functioning, role limitations due to physical health, role limitations due to emotional health, energy/fatigue, emotional well-being, social functioning, and pain [22]. Data for the general health domain were excluded due to a technical error during data transfer. Statistically significant increases in the scores for all domains except the pain domain were recorded during the observation period. Further analysis, stratified by self-reported adherence to therapy (low, moderate, or high), indicated that these improvements in HRQoL were restricted to patients with high compliance (data not shown; the number of respondents ranged from 21 to 35 for each question).

**Table 5:** HRQoL data accrued from patients who contributed to the digital data–collection element of DIAPAsOn. Differences (visit 2 vs visit 1 and visit 3 vs visit 1) were paired and therefore estimated only for those patients who were scored on both relevant visits. Data for the general health domain were excluded due to a technical error during data transfer.

			PF^a^		RP^b^		RE^c^		E/F^d^		EW^e^		SF^f^		P^g^
**Visit 1**															
	Respondents, n	82		82		82		82		82		82		82	
	Mean (SD) score	22.36 (16.18)		39.02 (44.11)		53.25 (41.2)		43.82 (21.62)		52.9 (16.1)		58.54 (26.34)		74.45 (31.16)	
**Visit 2**															
	Respondents, n	51		50		50		49		49		50		49	
	Mean (SD) score	34.56 (12.34)		87.5 (29.99)		93.33 (20.2)		72.28 (14.09)		76.01 (15.7)		89.25 (17.31)		94.23 (13.19)	
**Visit 2 vs visit 1**															
	Respondents, n	35		35		35		35		35		35		35	
	Mean (SD) score	7.63 (13.4)		32.86 (48.42)		25.71 (32.42)		20.81 (14.12)		22.66 (14.69)		26.43 (25.68)		8.43 (14.12)	
	*P* value	.002		<.001		<.001		<.001		<.001		<.001		.001	
**Visit 3**															
	Respondents, n	22		22		22		22		22		22		22	
	Mean (SD) score	40.1 (6.42)		98.86 (5.33)		100 (0)		80.23 (5.87)		86.73 (7.94)		98.3 (8)		98.52 (6.93)	
**Visit 3 vs visit 1**															
	Respondents, n	22		22		22		22		22		22		22	
	Mean (SD) score	6.69 (10.08)		29.55 (43.39)		18.18 (30.39)		18.18 (13.59)		25 (14.65)		16.48 (25.41)		-0.11 (0.53)	
	*P* value	.005		.004		.01		<.001		<.001		.006		.33	

^a^PF: physical functioning.

^b^RP: role limitations due to physical health.

^c^RE: role limitations due to emotional health.

^d^E/F: energy/fatigue.

^e^EW: emotional well-being.

^f^SF: social functioning.

^g^P: pain.

### Safety and Adverse Events Data

The safety population included all patients who had completed at least visit 1 (2572).

A total of 4 adverse drug reactions (ADRs) were recorded in 3 patients (0.12%). Two patients had 1 ADR and 1 patient had 2 ADRs. No serious ADRs were recorded during DIAPAsOn.

Four deaths were recorded during the study, including 1 from cardiovascular disease. None of the deaths were causally related to the use of OM3EE.

There were 20 instances of hospitalization due to cardiovascular diseases, none of which were attributed to the use of OM3EE. Thirteen of these events affected participants who were being treated for hypertriglyceridemia, all of whom were also being medicated with statins, fibrates, or both.

OM3EE therapy was discontinued by 69 patients. Specified reasons for doing so included inconvenience of use (11/69), lack of availability in pharmacies (6/69) and lack of effect (2/69). Reasons for the remaining 50 discontinuations were recorded as “other.”

## Discussion

### OM3EE Effect on Lipid Profile

Considered overall, the data from DIAPAsOn suggest that the introduction of OM3EE had favorable effects on the blood lipid profile of our patients, consistent with experiences in previous controlled trials. As illustrated in Table 3, the percentage of patients recorded as having TG <1.7 mmol/L quadrupled in response to OM3EE (from 282/2151, 13.1%, at baseline to 1028/1905, 54%, at the conclusion of the study); conversely, the percentage of patients recorded as having TG >2.3 mmol/L fell to 10.4% (198/1905) from 66.9% 1438/2151) at baseline. These changes were accompanied by alterations in other lipoprotein fractions compatible with an overall shift to a less atherogenic lipid profile, including a reduction in non-HDL-C, which declined by an average of 1.6 (SD 1.54) mmol/l. This pattern of response to OM3EE was substantially independent of the use or nonuse of statins by patients treated for hypertriglyceridemia (Table 4).

### Digital Versus Nondigital Adherence Findings

A central purpose of DIAPAsOn was to examine how the use of digital technologies might promote adherence to OM3EE therapy. This aspect of the study provided inconclusive and somewhat perplexing insights. The online facilities developed for DIAPAsOn were used by 180 of the 2167 patients (8.3%) in the analysis population.

Establishing why so many of our patients declined to use this option would require in-depth interviewing of several thousand people and is beyond the resources of the study as originally conceived. Similarly, we are not equipped to investigate whether or how physicians advocated for this aspect of the study during clinic visits or how patients might have responded to this encouragement. In retrospect, the lack of provision for detailed scrutiny of these matters is a limitation of our overall plan.

To a substantial (though unforeseen) extent, our study can be construed as an exploration of what may be called “spontaneous” adherence to a digital health initiative in response to an “open” invitation to a large and heterogeneous patient group. Our experience suggests that self-motivated engagement is exhibited by only a minority of patients. To the extent that this is a correct interpretation, it seems reasonable to conclude that plans to introduce such technologies need to place a much greater emphasis than we did on introducing and “selling” the concept and practice of eHealth to patients. The influences on engagement identified by Al-Naher et al [23] in their recent review of this field likely also applied to our study cohort; it must be acknowledged that limited emphasis was placed on these factors in our protocol. Many of the determinants of successful adoption of eHealth initiatives identified by Granja et al [24] will have been operative in the DIAPAsOn population (both patients and physicians). Notably, we may have made too little formal provision to anticipate and address patient concerns over privacy and security and physician concerns over workload.

Adherence to therapy for patients self-reporting via the DIAPAsOn digital platform was defined as the total number of days that a patient took the full prescribed dose of OM3EE during the specified period divided by the total number of days in that period. Calculating this way, adherence appeared to be low in these patients and declined during the period of observation. However, we have no means of ascertaining whether the data that the patients recorded accurately reflected their true adherence to study medication; actual adherence rates may therefore have been higher than the recorded findings suggest. This would be compatible with the finding that in-study trends in lipid and lipoprotein indices were favorable and numerically very similar in both digital adopters and the rest of the analysis population.

Comparison of the digital subset with the main analysis population identified no demographic differences between the two groups that might explain the adoption or nonadoption of the digital resources of DIAPAsOn. Wide-ranging technical obstacles seem unlikely given the high level of smartphone penetration in Russia [25], general access to the internet, and the requirement for digital proficiency as an inclusion criterion. The average age of the study population (approximately 58 years) is not, prima facie, a sufficient explanation for the low level of digital uptake but may have exerted an influence that our study was not calibrated to identify.

Seemingly at odds with the low level of adoption of the mobile technology devised for DIAPAsOn—and the apparently low levels of medication adherence reported by those patients that used the technology—is the observation that the dropout rate among the digital adopters was zero. The impression of a subset of patients who are tenacious in their adherence to technology but inattentive in their reported adherence to medications is a paradox that we are at present unable to rationalize.

Another finding of note was that the HRQoL indices in the digitally engaged patients showed a striking and sustained improvement among those with self-reported high compliance. This study had small patient numbers and had an observational design that precluded a determination of cause and effect. Thus, ascertaining reasons for the improvement in HRQoL indices lies outside the scope of our research. This is, nevertheless, an intriguing finding that would merit attention in future investigations.

### Features and Limitations of This Study

Mobile- or internet-based health interventions to promote adherence to therapy are considered to have potential, but to need enhanced quality and range of research [26-28]. Four aspects of DIAPAsOn should be examined in this context. First, our original intention was to conduct a study that emphasized inclusivity and a wide geographical distribution in order to, as we saw it, gain as much real-world (and by implication generalizable) experience as was possible with both the n-3 PUFA preparation and the digital engagement instruments. To that end, we applied what might, with the benefit of hindsight, be seen as an excessively “open” approach to recruitment: access to and proficiency with digital technology was a prerequisite for participation, but we did not explore with individual patients their a priori willingness to use such technology and to sustain that use over a period of several months. Second, instruction in the use of the technology was essentially delegated to individual investigators; they, while fully competent as clinicians and clinical researchers, may not have been best qualified to instruct, monitor, or motivate patients in this aspect of the study. We do not know the extent (if any) to which patients’ misunderstanding of what was being asked of them contributed to the outcome. Third, the digital facilities used in DIAPAsOn were developed in conjunction with a professional technology provider that has substantial experience and success in providing such services to the medical community in Russia. Much of that experience is at registry level, however, with input from physicians or trained assistants. We sought to make the technology accessible and frictionless to “retail” users, but the facts of our experience suggest that this aspect of our program may not have been successful. Here again, however, we are unable to say with assurance if that really was the case and, if so, why. Fourth, our work might perhaps have benefited from a small-scale scoping study or a pilot phase before the technology was deployed in such a large patient population. The work of Chen et al [29] with the Innovative Telemonitoring Enhanced Care Program for Chronic Heart Failure system provides a model of how to explore patient engagement in such a preliminary phase. Evaluation of our digital platform by means of the Mobile Application Rating Scale [30] might also have helped to refine the technology and enhance its acceptance by patients, and the failure to apply that test in advance may be considered a missed opportunity. One hazard of such an approach, however, is that by catering to the priorities of patients already well-disposed toward mobile health technology, the needs of “digital exiles” are overlooked. To that extent, DIAPAsOn has provided useful insights into the sort of real-world populations that might be encountered (at least in Russia) and some of the challenges that these populations pose for proponents of mobile or eHealth services. Provision for a more rigorous, ongoing interrogation of patients’ lack of compliance with the electronic facilities devised for this study would, with hindsight, have been prudent, as well as possibly informative, and we would advocate for such provision in any similar future research.

An overarching conclusion from this experience has to be that active patient (and physician) engagement and participation in the development of an online or mobile adherence aid is critical for successful longer-term adoption. With hindsight, the omission of such a stage from our study may be seen as a missed opportunity and is something we would prioritize in any similar future study.

The duration of follow up in DIAPAsOn was appropriate for a first assessment of a technical innovation in conjunction with an established therapy, but a substantially longer period of observation would be needed to demonstrate robust and meaningful improvements in long-term compliance and adherence, regardless of the technologies or medications used. This is also a consideration that we would factor into any future similar research projects.

As with observational studies in general, the absence of a control group precludes any determination of cause and effect, and the potential for biases in any trial of this type must be acknowledged. A retrospective calculation of the Nichol score [31] for DIAPAsOn confirmed that our study rated favorably in the subcategories “disease-related criteria” and “compliance definition and measurement criteria” but scored less strongly in the subcategory “study design criteria.”

### Conclusions

Uptake of digital methods for self-reporting adherence to therapy was low in this study and indicates a need for further research into the factors that motivate or discourage patients to take advantage of such services and how best to use these technologies to promote treatment compliance. Properly resourced attention to these considerations needs to be incorporated into study protocols.

Data collected through DIAPAsOn confirm the clinical profile of OM3EE as an effective and well-tolerated lipid-modifying therapy and as an appropriate element of a medical regime for the management of hypertriglyceridemia or the secondary prevention of MI. Substantial (approximately 1 mmol/L) baseline-dependent reductions in TG were recorded, and other nominally advantageous alterations in the lipid profile were apparent, including reduction in levels of non-HDL-C, regardless of the concomitant use of statins or fibrates. Investigation into compliance with therapy produced conflicting results, depending on the method of reporting used.

## Appendix

Multimedia Appendix 1 Listing of DIAPASoN center investigators.

Multimedia Appendix 2 Supplementary tables.

## Abbreviations

ADR: adverse drug reaction
CEPHEUS II: Centralized Pan-Russian Survey of the Undertreatment of Hypercholesterolemia II
HDL-C: high-density lipoprotein cholesterol
HRQoL: health-related quality of life
LDL-C: low-density lipoprotein cholesterol
MI: myocardial infarction
n-3 PUFA: omega-3 polyunsaturated fatty acid
OM3EE: omega-3-acid ethyl esters
TC: total cholesterol
TG: triglyceride
