# Supplementary material for: Use of Digital Technology Tools to Characterize Adherence to Prescription-Grade Omega-3 Polyunsaturated Fatty Acid Therapy in Postmyocardial or Hypertriglyceridemic Patients in the DIAPAsOn Study: Prospective Observational Study
Source: JMIR Cardio. 2022 Jul 25;6(2):e37490. doi: 10.2196/37490 (PMC9361151; doi:10.2196/37490)
Supplement: Multimedia Appendix 1 [file cardio_v6i2e37490_app1.docx]

*Appendix 1***.** Listing of DIAPASoN center investigators.

| Region | Investigator |
| --- | --- |
|  |  |
| Astrakhan Oblast | 1. Borodulya Mihail Vasil'evich |
| Bryansk Oblast | 1. Petuhova Irina Leonidovna |
| Vladimir Oblast | 1. Timofeeva Irina Vladimirovna |
| Volgograd Oblast | 1. Vorob'eva Svetlana Gennad'evna |
|  | 1. Gulova Ol'ga Aleksandrovna |
|  | 1. Dugencova Larisa Anatol'evna |
|  | 1. Sytilina Natal'ya Nikolaevna |
| Voronezh Oblast | 1. Trubicyna Irina Valentinovna |
| Kemerovo Oblast | 1. Krestova Ol'ga Sergeevna |
| Krasnodar Krai | 1. Alekseeva Elena Valer'evna |
|  | 1. Allaabed Hassan Adnanovich |
|  | 1. Bahmet'eva Irina Aleksandrovna |
|  | 1. Boldin Vasilij Borisovich |
|  | 1. Vikulova Larisa Vladimirovna |
|  | 1. Golovko Artyom Yur'evich |
|  | 1. Emuzova Liana Anatol'evna |
|  | 1. Zaharov Aleksandr Yur'evich |
|  | 1. Ivochkina Marina Ivanovna |
|  | 1. Imamutdinova Marina Vladimirovna |
|  | 1. Kovalenko Fyodor Andreevich |
|  | 1. Minasyan Ani Kamoevna |
|  | 1. Mkrtycheva Angelina Gennad'evna |
|  | 1. Nikitina Elena Valer'evna |
|  | 1. Novikova Svetlana Valer'evna |
|  | 1. Pavlovec Vadim Petrovich |
|  | 1. Perkova Elena Mihajlovna |
|  | 1. Prozorovskaya Yuliya Igorevna |
|  | 1. Ramenskaya Tat'yana Evgen'evna |
|  | 1. Raff Stanislav Anatol'evich |
|  | 1. Smolina Elena Garievna |
|  | 1. Subbotina Anastasiya Vladimirovna |
|  | 1. Tatarinceva Zoya Gennad'evna |
|  | 1. Usmanova Natal'ya Aleksandrovna |
|  | 1. Hazhbieva Milana Musaevna |
|  | 1. Shadzhe Evgeniya Azamatovna |
| Krasnoyarsk Krai | 1. Hamyt- Kyzy Ajperi Hamytovna |
| Moscow | 1. Adamyan Margarita Mamikonovna |
|  | 1. Belov Leonid L'vovich |
|  | 1. Brodichko Alena Ivanovna |
|  | 1. Bugaev Timofej Dmitrievich |
|  | 1. Vahrulina Natal'ya Konstantinovna |
|  | 1. Grigor'eva Ekaterina Anatol'evna |
|  | 1. Dvorina Ol'ga Gennad'evna |
|  | 1. Dmitrieva Irina Mihajlovna |
|  | 1. Karaeva Aida Anzorovna |
|  | 1. Novosel'ceva Ekaterina Pavlovna |
|  | 1. Oganesyan Lala Konstantinovna |
|  | 1. Polyakova Natal'ya Olegovna |
|  | 1. Ryzhova Tat'yana Vladimirovna |
|  | 1. Smirnova Ol'ga L'vovna |
|  | 1. Tavleeva Svetlana Nikolaevna |
|  | 1. Hrulenko Svetlana Borisovna |
|  | 1. Chernushenko Tat'yana Ivanovna |
| Moscow Oblast | 1. Gukov Konstantin Aleksandrovich |
|  | 1. Gukova Tat'yana Sergeevna |
|  | 1. Malyarenko Elena Nikolaevna |
|  | 1. Sorokin Sergej Anatol'evich |
| Nizhny Novgorod Oblast | 1. Abramova Nataliya Arkad'evna |
|  | 1. Aksenova Nataliya Aleksandrovna |
|  | 1. Barsukova Nataliya Aleksandrovna |
|  | 1. Budarina Yuliya Gennad'evna |
|  | 1. Grushin Dmitrij Valer'evich |
|  | 1. Kolesnichenko Irina Vyacheslavovna |
|  | 1. Lokonova Larisa Mihajlovna |
|  | 1. Fedorova Svetlana Nikolaevna |
| Novosibirsk Oblast | 1. Shurkevich Anastasiya Alekseevna |
| Omsk Oblast | 1. Minzhasarova Saniya Hasangalievna |
|  | 1. Naumov Dmitrij Valer'evich |
| Perm Krai | 1. Zhuravleva Natal'ya Alekseevna |
| The Republic of Adygea | 1. Shekhmirzova Dzhanetta Ruslanovna |
| The Republic of Bashkortostan | 1. Gilyaeva El'vira Fanisovna |
|  | 1. Dmitriev Aleksej Valer'evich |
|  | 1. Murasova Rimma Ismagilovna |
|  | 1. Tarzimanova Yuliya Shamilevna |
|  | 1. Timerbulatov Timur Rasfarovich |
| Tatarstan | 1. Ivanova Natal'ya Mihajlovna |
| Rostov Oblast | 1. Budanova Ol'ga Veniaminovna |
|  | 1. Lobe Aleksandra Ovanesovna |
|  | 1. Mazruho Marina Karpovna |
|  | 1. Morgun Nina Karpovna |
|  | 1. Stupina Anna Alekseevna |
| Ryazan Oblast | 1. Grusheckaya Irina Stanislavovna |
|  | 1. Samarceva Yana Nikolaevna |
| Samara Oblast | 1. Aristova Tat'yana Vladimirovna |
|  | 1. Reznik Irina Mihajlovna |
|  | 1. Rybina Evgeniya Dmitrievna |
|  | 1. Sapunkova Svetlana Mihajlovna |
|  | 1. Filippovskaya Natal'ya Igorevna |
|  | 1. Chernova Viktoriya Nikolaevna |
| Saint Petersburg | 1. Bulycheva-Samohina Lyubov' Vasil'evna |
|  | 1. Omel'chenko Marina Yur'evna |
|  | 1. Saf'yanova Natal'ya Viktorovna |
| Saratov Oblast | 1. Mihajlova Elena Aleksandrovna |
| Smolensk Oblast | 1. Novik Lyudmila Mihajlovna |
| Stavropol Krai | 1. Vedeneva Elena Viktorovna |
|  | 1. Eremenko Aleksej Mihajlovich |
|  | 1. Kubanova Asiyat Borisovna |
|  | 1. Minasova Elena Nikolaevna |
| Tomsk Oblast | 1. Zubova Ol'ga Valer'evna |
| Tula Oblast | 1. Barabanova Tat'yana Yur'evna |
|  | 1. Dabizha Viktoriya Gennad'evna |
|  | 1. Kolomejceva Tat'yana Mihajlovna |
|  | 1. Prihod'ko Tat'yana Nikolaevna |
| Tumen Oblast | 1. Ahshiyatova Nastya Ibragimovna |
| Khabarovsk Krai | 1. Koroleva Ramilya Lotfullovna |
| Chelyabinsk Oblast | 1. Malyutina Anastasiya Gennad'evna |
|  | 1. Fanina El'vira Rinatovna |
