# Supplementary material for: Use of Digital Technology Tools to Characterize Adherence to Prescription-Grade Omega-3 Polyunsaturated Fatty Acid Therapy in Postmyocardial or Hypertriglyceridemic Patients in the DIAPAsOn Study: Prospective Observational Study
Source: JMIR Cardio. 2022 Jul 25;6(2):e37490. doi: 10.2196/37490 (PMC9361151; doi:10.2196/37490)
Supplement: Multimedia Appendix 2 [file cardio_v6i2e37490_app2.docx]

*Supplementary Tables*

**Table** **S 1a. Assessment of the relationship between the rate of adherence and sex.**

| **Visit** | **Parameter** | **Gender** | | | |
| --- | --- | --- | --- | --- | --- |
|  |  | **Male** | | **Female** | |
|  |  | **N** | **Proportion (%)** | **N° Pt** | **Rate (%)** |
| Visit 1 – Visit 3 | Total | 1039 | 100 | 936 | 100 |
|  | Low adherence | 1 | 0.1 | 7 | 0.75 |
|  | Moderate adherence | 52 | 5 | 83 | 8.87 |
|  | High adherence | 180 | 17.32 | 214 | 22.86 |
|  | Very high adherence | 806 | 77.57 | 632 | 67.52 |
|  | Mean questionnaire score | 13.23 | | 12.53 | |
|  | p-value^*^ | <0.001 | | | |

Note: data are of the PP population, N = 2167; ^*^χ-square.

**Table S1b. Assessment of the relationship between the rate of adherence to therapy and the sex** **of patients to whom OM3EE was prescribed as secondary preventive therapy after myocardial infarction.**

| **Visit** | **Parameter** | **Gender** | | | |
| --- | --- | --- | --- | --- | --- |
|  |  | **Male** | | **Female** | |
|  |  | **N** | **Proportion (%)** | **N° Pt** | **Rate (%)** |
| Visit 1 – Visit 3 | Total | 528 | 100 | 252 | 100 |
|  | Low adherence | 0 | 0 | 0 | 0 |
|  | Moderate adherence | 10 | 1.89 | 7 | 2.78 |
|  | High adherence | 58 | 10.98 | 26 | 10.32 |
|  | Very high adherence | 460 | 87.12 | 219 | 86.9 |
|  | Mean questionnaire score | 13.9 | | 13.9 | |
|  | p-value^*^ | 0.98 | | | |

Note: data are of the PP population, N = 2167; ^*^χ-square.

**Table S1c. Assessment of the relationship between the rate of adherence to therapy and sex among patients to whom OM3EE was prescribed for hypertriglyceridemia**

| **Visit** | **Parameter** | **Gender** | | | |
| --- | --- | --- | --- | --- | --- |
|  |  | **Male** | | **Female** | |
|  |  | **N** | **Proportion (%)** | **N** | **Proportion (%)** |
| Visit 1 – Visit 3 | Total | 511 | 100 | 684 | 100 |
|  | Low adherence | 1 | 0.2 | 7 | 1.02 |
|  | Moderate adherence | 42 | 8.22 | 76 | 11.11 |
|  | High adherence | 122 | 23.87 | 188 | 27.49 |
|  | Very high adherence | 346 | 67.71 | 413 | 60.38 |
|  | Mean questionnaire score | 12.54 | | 12.03 | |
|  | p-value^*^ | 0.007 | | | |

Note: data are of the PP population, N = 2167; ^*^χ-square.

**Table S2a.** **Assessment of the relationship between the rate of adherence and age.**

| **Visit** | **Parameter** | **Age group** | | | | | | | |
| --- | --- | --- | --- | --- | --- | --- | --- | --- | --- |
|  |  | **18-44** | | **45-60** | | **61-75** | | **>75** | |
|  |  | **N** | **Proportion (%)** | **N** | **Proportion (%)** | **N** | **Proportion (%)** | **N** | **Proportion (%)** |
| Visit 1 – Visit 3 | Total | 231 | 100 | 768 | 100 | 804 | 100 | 172 | 100 |
|  | Low adherence | 1 | 0.43 | 3 | 0.39 | 4 | 0.5 | 0 | 0 |
|  | Moderate adherence | 21 | 9.09 | 52 | 6.77 | 48 | 5.97 | 14 | 8.14 |
|  | High adherence | 55 | 23.81 | 167 | 21.74 | 137 | 17.04 | 35 | 20.35 |
|  | Very high adherence | 154 | 66.67 | 546 | 71.09 | 615 | 76.49 | 123 | 71.51 |
|  | Mean questionnaire score | 12.45 | | 12.78 | | 13.15 | | 12.87 | |
|  | p-value^*^ | 0.007 | | | | | | | |

Note: data are of the PP population, N = 2167; ^*^χ-square.

**Table S2b. Assessment of the relationship between the rate of adherence to therapy and the age of patients to whom OM3EE was prescribed as secondary preventive therapy after myocardial infarction.**

| **Visit** | **Parameter** | **Age group** | | | | | | | |
| --- | --- | --- | --- | --- | --- | --- | --- | --- | --- |
|  |  | **18-44** | | **45-60** | | **61-75** | | **>75** | |
|  |  | **N** | **Proportion (%)** | **N** | **Proportion (%)** | **N** | **Proportion (%)** | **N** | **Proportion (%)** |
| Visit 1 – Visit 3 | Total | 48 | 100 | 273 | 100 | 378 | 100 | 81 | 100 |
|  | Low adherence | 0 | 0 | 0 | 0 | 0 | 0 | 0 | 0 |
|  | Moderate adherence | 1 | 2.08 | 3 | 1.1 | 8 | 2.12 | 5 | 6.17 |
|  | High adherence | 5 | 10.42 | 25 | 9.16 | 39 | 10.32 | 15 | 18.52 |
|  | Very high adherence | 42 | 87.5 | 245 | 89.74 | 331 | 87.57 | 61 | 75.31 |
|  | Mean questionnaire score | 13.92 | | 14.06 | | 13.95 | | 13.14 | |
|  | p-value^*^ | 0.007 | | | | | | | |

Note: data are of the PP population, N = 2167; ^*^χ-square.

**Table S2c. Assessment of the relationship between the rate of adherence to therapy and age among patients to whom OM3EE was prescribed for hypertriglyceridemia.**

| **Visit** | **Parameter** | **Age group** | | | | | | | |
| --- | --- | --- | --- | --- | --- | --- | --- | --- | --- |
|  |  | **18-44** | | **45-60** | | **61-75** | | **>75** | |
|  |  | **N** | **Proportion (%)** | **N** | **Proportion (%)** | **N** | **Proportion (%)** | **N** | **Proportion (%)** |
| Visit 1 – Visit 3 | Total | 183 | 100 | 495 | 100 | 426 | 100 | 91 | 100 |
|  | Low adherence | 1 | 0.55 | 3 | 0.61 | 4 | 0.94 | 0 | 0 |
|  | Moderate adherence | 20 | 10.93 | 49 | 9.9 | 40 | 9.39 | 9 | 9.89 |
|  | High adherence | 50 | 27.32 | 142 | 28.69 | 98 | 23 | 20 | 21.98 |
|  | Very high adherence | 112 | 61.2 | 301 | 60.81 | 284 | 66.67 | 62 | 68.13 |
|  | Mean questionnaire score | 12.07 | | 12.07 | | 12.44 | | 12.64 | |
|  | p-value^*^ | 0.19 | | | | | | | |

Note: data are of the PP population, N = 2167; ^*^χ-square.

**Table S3a. Assessment of the relationship between the rate of adherence and work status.**

| **Visit** | **Parameter** | **Work status** | | | |
| --- | --- | --- | --- | --- | --- |
|  |  | **Works** | | **Does not work** | |
|  |  | **N** | **Proportion (%)** | **N** | **Proportion (%)** |
| Visit 1 – Visit 3 | Total | 1096 | 100 | 879 | 100 |
|  | Low adherence | 5 | 0.46 | 3 | 0.34 |
|  | Moderate adherence | 85 | 7.76 | 50 | 5.69 |
|  | High adherence | 251 | 22.9 | 143 | 16.27 |
|  | Very high adherence | 755 | 68.89 | 683 | 77.7 |
|  | Mean questionnaire score | 12.66 | | 13.2 | |
|  | p-value^*^ | <0.001 | | | |

Note: data are of the PP population, N = 2167; ^*^χ-square.

**Table S3b. Assessment of the relationship between the rate of adherence to therapy and work status of patients to whom OM3EE was prescribed as secondary preventive therapy after myocardial infarction.**

| **Visit** | **Parameter** | **Work status** | | | |
| --- | --- | --- | --- | --- | --- |
|  |  | **Works** | | **Does not work** | |
|  |  | **N** | **Proportion (%)** | **N** | **Proportion (%)** |
| Visit 1 – Visit 3 | Total | 382 | 100 | 398 | 100 |
|  | Low adherence | 0 | 0 | 0 | 0 |
|  | Moderate adherence | 6 | 1.57 | 11 | 2.76 |
|  | High adherence | 47 | 12.3 | 37 | 9.3 |
|  | Very high adherence | 329 | 86.13 | 350 | 87.94 |
|  | Mean questionnaire score | 13.9 | | 13.9 | |
|  | p-value^*^ | 0.99 | | | |

Note: data are of the PP population, N = 2167; ^*^χ-square.

**Table S3c. Assessment of the relationship between the rate of adherence to therapy and work status among patients to whom OM3EE was prescribed for hypertriglyceridemia.**

| **Visit** | **Parameter** | **Work status** | | | |
| --- | --- | --- | --- | --- | --- |
|  |  | **Works** | | **Does not work** | |
|  |  | **N** | **Proportion (%)** | **N** | **Proportion (%)** |
| Visit 1 – Visit 3 | Total | 714 | 100 | 481 | 100 |
|  | Low adherence | 5 | 0.7 | 3 | 0.62 |
|  | Moderate adherence | 79 | 11.06 | 39 | 8.11 |
|  | High adherence | 204 | 28.57 | 106 | 22.04 |
|  | Very high adherence | 426 | 59.66 | 333 | 69.23 |
|  | Mean questionnaire score | 11.99 | | 12.62 | |
|  | p-value^*^ | 0.001 | | | |

Note: data are of the PP population, N = 2167; ^*^χ-square.

**Table S4a. Assessment of the relationship between the rate of adherence and education.**

| **Visit** | **Parameter** | **Education** | | | | | | | | | |
| --- | --- | --- | --- | --- | --- | --- | --- | --- | --- | --- | --- |
|  |  | **Secondary education drop-out** | | **Secondary general** | | **Secondary vocational** | | **Higher** | | **Supplementary vocational** | |
|  |  | **N** | **Proportion (%)** | **N** | **Proportion (%)** | **N** | **Proportion (%)** | **N** | **Proportion (%)** | **N** | **Proportion (%)** |
| Visit 1 – Visit 3 | Total | 15 | 100 | 224 | 100 | 634 | 100 | 1064 | 100 | 38 | 100 |
|  | Low adherence | 0 | 0 | 3 | 1.34 | 3 | 0.47 | 2 | 0.19 | 0 | 0.19 |
|  | Moderate adherence | 0 | 0 | 24 | 10.71 | 56 | 8.83 | 52 | 4.89 | 3 | 4.89 |
|  | High adherence | 1 | 6.67 | 67 | 29.91 | 121 | 19.09 | 192 | 18.05 | 13 | 18.05 |
|  | Very high adherence | 14 | 93.33 | 130 | 58.04 | 454 | 71.61 | 818 | 76.88 | 22 | 76.88 |
|  | Mean questionnaire score | 14.27 | | 11.91 | | 12.67 | | 13.25 | | 12.21 | |
|  | p-value^*^ | <0.001 | | | | | | | | | |

Note: data are of the PP population, N = 2167; ^*^χ-square.

**Table S4b. Assessment of the relationship between the rate of adherence to therapy and education of patients to whom OM3EE was prescribed as secondary preventive therapy after myocardial infarction.**

| **Visit** | **Parameter** | **Education** | | | | | | | | | |
| --- | --- | --- | --- | --- | --- | --- | --- | --- | --- | --- | --- |
|  |  | **Secondary education drop-out** | | **Secondary general** | | **Secondary vocational** | | **Higher** | | **Supplementary vocational** | |
|  |  | **N** | **Proportion (%)** | **N** | **Proportion (%)** | **N** | **Proportion (%)** | **N** | **Proportion (%)** | **N** | **Proportion (%)** |
| Visit 1 – Visit 3 | Total | 12 | 100 | 78 | 100 | 258 | 100 | 419 | 100 | 13 | 100 |
|  | Low adherence | 0 | 0 | 0 | 0 | 0 | 0 | 0 | 0 | 0 | 0 |
|  | Moderate adherence | 0 | 0 | 3 | 3.85 | 8 | 3.1 | 6 | 1.43 | 0 | 1.43 |
|  | High adherence | 1 | 8.33 | 12 | 15.38 | 24 | 9.3 | 46 | 10.98 | 1 | 10.98 |
|  | Very high adherence | 11 | 91.67 | 63 | 80.77 | 226 | 87.6 | 367 | 87.59 | 12 | 87.59 |
|  | Mean questionnaire score | 14.17 | | 13.46 | | 13.84 | | 14.01 | | 14.15 | |
|  | p-value^*^ | 0.3 | | | | | | | | | |

Note: data are of the PP population, N = 2167; ^*^χ-square.

**Table S4c. Assessment of the relationship between the rate of adherence to therapy and education among patients to whom OM3EE was prescribed for hypertriglyceridemia.**

| **Visit** | **Parameter** | **Education** | | | | | | | | | |
| --- | --- | --- | --- | --- | --- | --- | --- | --- | --- | --- | --- |
|  |  | **Secondary education drop-out** | | **Secondary general** | | **Secondary vocational** | | **Higher** | | **Supplementary vocational** | |
|  |  | **N** | **Proportion (%)** | **N** | **Proportion (%)** | **N** | **Proportion (%)** | **N** | **Proportion (%)** | **N** | **Proportion (%)** |
| Visit 1 – Visit 3 | Total | 3 | 100 | 146 | 100 | 376 | 100 | 645 | 100 | 25 | 100 |
|  | Low adherence | 0 | 0 | 3 | 2.05 | 3 | 0.8 | 2 | 0.31 | 0 | 0.31 |
|  | Moderate adherence | 0 | 0 | 21 | 14.38 | 48 | 12.77 | 46 | 7.13 | 3 | 7.13 |
|  | High adherence | 0 | 0 | 55 | 37.67 | 97 | 25.8 | 146 | 22.64 | 12 | 22.64 |
|  | Very high adherence | 3 | 100 | 67 | 45.89 | 228 | 60.64 | 451 | 69.92 | 10 | 69.92 |
|  | Mean questionnaire score | 14.67 | | 11.08 | | 11.88 | | 12.76 | | 11.2 | |
|  | p-value^*^ | <0.001 | | | | | | | | | |

Note: data are of the PP population, N = 2167; ^*^χ-square.

**Table S5a. Assessment of the relationship between the rate of adherence and marital status.**

| **Visit** | **Parameter** | **Marital status** | | | | | | | |
| --- | --- | --- | --- | --- | --- | --- | --- | --- | --- |
|  |  | **Single** | | **Married** | | **Divorced** | | **Widowed** | |
|  |  | **N** | **Proportion (%)** | **N** | **Proportion (%)** | **N** | **Proportion (%)** | **N** | **Proportion (%)** |
| Visit 1 – Visit 3 | Total | 82 | 100 | 1593 | 100 | 114 | 100 | 186 | 100 |
|  | Low adherence | 0 | 0 | 7 | 0.44 | 1 | 0.88 | 0 | 0 |
|  | Moderate adherence | 13 | 15.85 | 102 | 6.4 | 8 | 7.02 | 12 | 6.45 |
|  | High adherence | 13 | 15.85 | 320 | 20.09 | 24 | 21.05 | 37 | 19.89 |
|  | Very high adherence | 56 | 68.29 | 1164 | 73.07 | 81 | 71.05 | 137 | 73.66 |
|  | Mean questionnaire score | 12.5 | | 12.91 | | 12.76 | | 13.08 | |
|  | p-value^*^ | 0.49 | | | | | | | |

Note: data are of the PP population, N = 2167; ^*^χ-square.

**Table S5b. Assessment of the relationship between the rate of adherence to therapy and marital status** **of patients** **to whom OM3EE was prescribed as secondary preventive therapy after myocardial infarction.**

| **Visit** | **Parameter** | **Marital status** | | | | | | | |
| --- | --- | --- | --- | --- | --- | --- | --- | --- | --- |
|  |  | **Single** | | **Married** | | **Divorced** | | **Widowed** | |
|  |  | **N** | **Proportion (%)** | **N** | **Proportion (%)** | **N** | **Proportion (%)** | **N** | **Proportion (%)** |
| Visit 1 – Visit 3 | Total | 31 | 100 | 574 | 100 | 57 | 100 | 118 | 100 |
|  | Low adherence | 0 | 0 | 0 | 0 | 0 | 0 | 0 | 0 |
|  | Moderate adherence | 4 | 12.9 | 7 | 1.22 | 3 | 5.26 | 3 | 2.54 |
|  | High adherence | 6 | 19.35 | 55 | 9.58 | 5 | 8.77 | 18 | 15.25 |
|  | Very high adherence | 21 | 67.74 | 512 | 89.2 | 49 | 85.96 | 97 | 82.2 |
|  | Mean questionnaire score | 12.58 | | 14.01 | | 13.79 | | 13.8 | |
|  | p-value^*^ | 0.004 | | | | | | | |

Note: data are of the PP population, N = 2167; ^*^χ-square.

**Table S5c. Assessment of the relationship between the rate of adherence to therapy and marital status among patients to whom OM3EE was prescribed for hypertriglyceridemia.**

| **Visit** | **Parameter** | **Marital status** | | | | | | | |
| --- | --- | --- | --- | --- | --- | --- | --- | --- | --- |
|  |  | **Single** | | **Married** | | **Divorced** | | **Widowed** | |
|  |  | **N** | **Proportion (%)** | **N** | **Proportion (%)** | **N** | **Proportion (%)** | **N** | **Proportion (%)** |
| Visit 1 – Visit 3 | Total | 51 | 100 | 1019 | 100 | 57 | 100 | 68 | 100 |
|  | Low adherence | 0 | 0 | 7 | 0.69 | 1 | 1.75 | 0 | 0 |
|  | Moderate adherence | 9 | 17.65 | 95 | 9.32 | 5 | 8.77 | 9 | 13.24 |
|  | High adherence | 7 | 13.73 | 265 | 26.01 | 19 | 33.33 | 19 | 27.94 |
|  | Very high adherence | 35 | 68.63 | 652 | 63.98 | 32 | 56.14 | 40 | 58.82 |
|  | Mean questionnaire score | 12.45 | | 12.29 | | 11.74 | | 11.84 | |
|  | p-value^*^ | 0.42 | | | | | | | |

Note: data are of the PP population, N = 2167; ^*^χ-square.
